# YY1 mediated DCUN1D5 transcriptional activation promotes triple-negative breast cancer progression by targeting FN1/PI3K/AKT pathway

**DOI:** 10.1186/s13062-024-00481-2

**Published:** 2024-06-03

**Authors:** Yuxiang Lin, Yan Li, Xiaobin Chen, Xuan Jin, Meichen Jiang, Han Xiao, Lili Chen, Minyan Chen, Wenzhe Zhang, Hanxi Chen, Qian Nie, Rongrong Guo, Wenhui Guo, Fangmeng Fu, Chuan Wang

**Affiliations:** 1 Department of Breast Surgery, Fujian Medical University Union Hospital, No.29, Xin Quan Road, Gulou District, Fuzhou, 350001 Fujian Province China; 2 Department of General Surgery, Fujian Medical University Union Hospital, Fuzhou, 350001 Fujian Province China; 3 Breast Cancer Institute, Fujian Medical University, Fuzhou, Fujian Province China; 4 Department of Pathology, Fujian Medical University Union Hospital, Fuzhou, 350001 Fujian Province China

**Keywords:** Triple-negative breast cancer, Progression, DCUN1D5, FN1, YY1

## Abstract

Triple-negative breast cancer (TNBC) is more aggressive and has a higher metastasis rate compared with other subtypes of breast cancer. Due to the lack of drug-targetable receptors, chemotherapy is now the only available systemic treatment for TNBC. However, some patients might still develop drug resistance and have poor prognosis. Therefore, novel molecular biomarkers and new treatment targets are urgently needed for patients with TNBC. To provide molecular insights into TNBC progression, we investigated the function and the underlying mechanism of Defective in cullin neddylation 1 domain containing 5 (DCUN1D5) in the regulation of TNBC. By TCGA dataset and surgical specimens with immunohistochemical (IHC) staining method, DCUN1D5 was identified to be significantly upregulated in TNBC tumor tissues and negatively associated with prognosis. A series of in vitro and in vivo experiments were performed to confirm the oncogenic role of DCUN1D5 in TNBC. Overexpression of FN1 or PI3K/AKT activator IGF-1 could restore the proliferative and invasive ability induced by DCUN1D5 knockdown and DCUN1D5 could act as a novel transcriptional target of transcription factor Yin Yang 1 (YY1). In conclusion, YY1-enhanced DCUN1D5 expression could promote TNBC progression by FN1/PI3K/AKT pathway and DCUN1D5 might be a potential prognostic biomarker and therapeutic target for TNBC treatment.

**Supplementary Information:**

The online version contains supplementary material available at 10.1186/s13062-024-00481-2.

## Background

Breast cancer (BC) is the most frequently diagnosed cancer in women, accounting for about 10% of all new malignancies worldwide [1]. Triple-negative breast cancer (TNBC) is a subtype of breast cancer characterized by the absence of estrogen receptor (ER), progesterone receptor (PR) and human epidermal growth factor 2 (HER2) expression. Compared with other subtypes of breast cancer, TNBC is more aggressive and has a higher metastasis rate [2, 3]. Due to the lack of drug-targetable receptors, chemotherapy is now the only available systemic treatment for TNBC [4]. However, some patients might still develop drug resistance and have poor prognosis [5]. Therefore, novel molecular biomarkers and new treatment targets are urgently needed for patients with TNBC.

Defective in cullin neddylation 1 domain containing 1–5 (also known as squamous cell carcinoma related oncogene 1–5, SCCRO1-5) are a group of protein family which could act as key components of the E3 for neddylation [6]. These DCUN1Ds have distinct N-terminal regions, but they share a conserved C-terminal potentiating neddylation (PONY) domain. DCUN1D1-DCUN1D5 paralogues are subdivided into three subfamilies on the basis of the N-terminal sequence. DCUN1D1 and DCUN1D2 contain a ubiquitin-associated (UBA) domain, DCUN1D3 contains a myristoylation sequence, while DCUN1D4 and DCUN1D5 contain a nuclear localization sequence (NLS) [7–9]. The regulation of DCUN1D activity has been shown to play important roles in tumorigenesis. For instance, DCUN1D1 and DCUN1D3 were reported to be highly expressed in numerous tumors and could act as oncogenes [10–13]. As for DCUN1D5, it has also been proved to be associated with unfavorable prognosis and could promote the proliferation of oral and lung squamous cell carcinomas [7, 14]. In breast cancer, DCUN1D5 was identified to be highly expressed in metastatic breast tumors, especially for TNBC subtype [15]. Nevertheless, the oncogenic function of DCUN1D5 in TNBC remains still largely unknown.

In the current investigation, we reported that DCUN1D5 was highly expressed and correlated with a poor survival in patients with TNBC. Functional studies indicated that DCUN1D5 could serve as an oncogene in TNBC by activating the FN1/PI3K/AKT pathway. Moreover, DCUN1D5 was also identified as a novel transcriptional target of YY1. Taken together, these findings provide novel insights of DCUN1D5 in the progression of TNBC and suggest a new theoretical basis for the treatment of triple-negative breast cancer.

## Methods

### Microarray data collection and patient specimens

The mRNA microarray dataset with TNBC patients were extracted from the Cancer Genome Atlas (TCGA) database (https://portal.gdc.cancer.gov/) [16]. Moreover, a total of 166 paired paraffin embedding TNBC specimens and corresponding non-tumor tissues were obtained from the Fujian Medical University Union Hospital from January 2017 to April 2019. The clinical and follow-up information regarding each patient was collected. Disease-free survival (DFS) was defined as the time from the date of diagnosis to the date of relapse/metastasis. Overall survival (OS) was defined as the time from the date of diagnosis until death from any cause. The follow-up deadline was April 1, 2022. This procedure was approved by the Research Ethics Committee of Fujian Medical University Union Hospital. Informed consent was obtained from each participant.

### Immunohistochemistry (IHC) staining

IHC staining analysis was performed to measure the protein expression of DCUN1D5 in TNBC tissues and corresponding non-tumor tissues according to the standard staining procedure. Slides were incubated with anti-DCUN1D5 (1:400; 14810-1-AP, Proteintech) according to the manufacturer’s instructions. The IHC staining scores were calculated as product of the proportion and intensity for the stained tumor cells. The proportion of stained positive cells was scored from 1 to 4: 1, 0–25%; 2, 26–50%; 3, 51–75%; and 4, 75–100%, while the staining intensity score was from 0 to 3: 0, no staining; 1, weak staining; 2, moderate staining; and 3, strong staining. For each patient, a score of 8–12 was defined as high expression level and a score of 0–7 was defined as low expression level. The IHC staining scores were evaluated by two independent pathologists blinded to each patient.

### Cell culture and transfection

Human triple-negative breast cancer cell lines (MDA-MB-231, BT-549, MDA-MB-468, Hs578T) and non-tumorigenic breast epithelial cell line (MCF-10 A) were purchased from the Cell Bank of Type Culture Collection of The Chinese Academy of Sciences (Shanghai, China) in May 2021. All cell lines were cultured in DMEM (HyClone) containing 10% FBS (Gibco), 1% penicillin and streptomycin solution in an incubator with standard condition (5% CO_2_, 37 °C). The genetic testing and identification of the cell lines described in this study was conducted by the short tandem repeat (STR) profiling method. The cell lines selected for experimentation had been identified within a timeframe of less than 16 weeks. The most recent identification was carried out in June 2023. Short hairpin RNA (shRNA) targeting DCUN1D5 were subcloned into GV493 lentiviral shRNA vector (Genechem, Shanghai, China). For overexpressing DCUN1D5, FN1 and YY1, the construct was generated by subcloning PCR amplified full-length human cDNA. The constructed lentiviral vectors were packaged into the viruses in HEK 293 T cells. Then, the harvested and concentrated viruses were added into cells and cultured for 72 h. The target sequences of the shDCUN1D5 and shCtrl were as follows:

shDCUN1D5-1: 5’-ATGCTGATCTTAGTAACTA-3’.

shDCUN1D5-2: 5’-GCTAGACTAATAAGTGGAGAG-3’.

shDCUN1D5-3: 5’-GCAGGTCCTGATGAAGTTGTA-3’.

shCtrl: 5’-TTCTCCGAACGTGTCACGT-3’.

### Quantitative real-time PCR

Total RNA was extracted from different cell lines with TRIzol reagent (Invitrogen). Reverse transcription of cDNA was performed by PrimeScript RT Master Mix (Takara) and qRT-PCR was subsequently carried out following the instructions of the SYBR Green Real-Time PCR kit with an ABI 7500 PCR System (Applied Biosystems). The relative expression of the target genes was normalized to GAPDH and data analyses were calculated by the 2 − ΔΔct method. The primers used in qRT-PCR were shown in Table S1.

### Western blot analysis

Total protein was separated by RIPA lysis buffer (Beyotime) and quantified with BCA Protein Assay Kits (Beyotime). Protein samples were probed with the following antibodies: anti-DCUN1D5 (14810-1-AP, Proteintech), anti-FN1 (#26,836, Cell Signaling Technology), anti-p-PI3K (20584-1-AP, Proteintech), anti-PI3K (ab154598, abcam), anti-p-AKT (28731-1-AP, Proteintech), anti-AKT (10176-2-AP, Proteintech) and anti-GAPDH (ab181602, Abcam). Binding of the primary antibody was detected by incubating the membranes with a horseradish peroxidase-conjugated secondary antibody. Immunoblots were observed with the Enhanced Chemiluminescence Detection Kit (Thermo Fisher Scientific, Inc.).

### Cell proliferation and colony formation assays

Cell proliferation was assessed by CCK8 assay, in which cells were seeded in 96-well plates (2000 cells/well). Then, 10 ul of Cell Counting Kit-8 (Dojindo Molecular Technologies) was added to each well and incubated for 2 h at 37 °C. The absorbance was measured at 450 nm through a microplate reader. Cell growth was analyzed once per day for 5 days. For the colony formation assay, 1000 cells were seeded into a 6-well plate and cultured at 37 °C for 14 days. At the end of the incubation, colonies were fixed with 4% paraformaldehyde for 30 min and stained with 0.1% of crystal violet solution (Sangon Bio, Inc.) for 15 min.

### Apoptosis assay

Apoptosis assay were performed with the Annexin V-APC Apoptosis Detection Kit (eBioscience) according to the manufacturer’s instructions, then the apoptosis rate was evaluated by a flow cytometer (Guava EasyCyte HT).

### Wound healing and transwell assays

For wound healing assay, cells were seeded at the bottom of 6-well plates and cultured at 37 °C overnight. A 10 ul sterile tip was used to produce a line wound across the confluent cell layer. The floating cells were washed with PBS and the serum-free medium was utilized to maintain the cells. The images were captured at 0 and 24 h, then cell migration was calculated through the healed wound percentage. Transwell assays were performed to detect cell migration and invasion, the upper chamber of the transwell (Corning, Inc.) was filled with 100 μl of cell suspension, and the lower chamber was filled with 600 μl of DMEM with 30% FBS. For invasion assays, transwell chambers were precoated with Matrigel (BD Biosciences) for 2 h at 37 °C. After 24 h, the migrated or invaded cells were fixed with 4% paraformaldehyde and stained with 0.5% crystal violet. The images of stained cells on the lower side were captured.

### Tumor formation and metastasis assays in nude mouse models

In animal study, 4-week-old female BALB/c nude mice were purchased from Vital River Co. Ltd. (Beijing, China) and kept in sterile cages with a specific nonpathogenic condition. For xenograft animal models, the mice were randomly divided into two groups (*n* = 10 for each group). A total of 1 × 10^7^ shDCUN1D5 MDA-MB-231 cells or negative control (shCtrl) cells were subcutaneously injected into the BALB/c nude mice. Tumor volume was recorded every 3 days and calculated according to the formula: (length × width^2^)/2. After 25 days of injection, all mice were sacrificed and the xenografts were dissected and weighed. IHC staining analysis was conducted to measure the expression of DCUN1D5, Ki67, FN1, p-PI3K and p-AKT of xenograft tumors. The antibodies used in the IHC staining analysis was listed in Table S2. For the in vivo lung metastasis assay, shDCUN1D5 MDA-MB-231 or shCtrl cells (2 × 10^6^) were injected into the tail vein of each nude mouse (*n* = 5 for each group). These mice were sacrificed after 10 weeks and the lungs were resected for H&E staining to calculate metastatic nodules. Two consecutive 5 μm sections were taken and then a number of consecutive 5 μm sections were discarded before collecting another two consecutive 5 μm sections. This process was repeated along the entire lung. Metastatic nodules were counted on each H&E paraffin section with a phase contrast microscope and the sum of microscopic counting were adopted as the final number of lung metastatic nodules. All animal experiments were conducted with the approval by the Animal Ethics Committee of Fujian Medical University. Relevant procedures were performed in accordance with the guidelines for the care and welfare of laboratory animals.

### RNA-seq

RNA-seq was performed to detect the mRNA expression profiles of DCUN1D5 knockdown MDA-MB-231 cells (shDCUN1D5) and negative control (shCtrl). Total RNA was extracted and controlled for quality by a BioAnalyzer 2100 system (Agilent Technologies, Inc.). Then, RNA-seq was subsequently performed by Illumina NovaSeq™ 6000 platform. The sequencing data were mapping to the reference genome with HISAT2 (v2.0.5) and differential expression analysis was performed by the DESeq2(v1.16.1).

### Dual-luciferase reporter assay

The human DCUN1D5 promoter fragment within WT or Mut binding site was cloned into a GV238 vector to perform luciferase activity assays. Cells were seeded in 24-well plates and transfected with relevant plasmids and the luciferase vector. After 48 h, luciferase activity was detected by Promega Luciferase Reporter Assay System.

### Chromatin immunoprecipitation (ChIP) assay

The ChIP assay was performed with the EZ ChIP™ Chromatin Immunoprecipitation Kit (Millipore) in accordance with the manufacturer’s instructions. The antibodies used in ChIP assay were anti-YY1 (#63,227, Cell Signaling Technology) and normal IgG (#2729, Cell Signaling Technology). The enriched DNA was analyzed by real-time PCR.

### Statistical analysis

The correlations between DCUN1D5 expression and the clinicopathological parameters were evaluated by chi-Square test. Survival curves were analyzed by Kaplan-Meier method and compared by log-rank test. Cox proportional hazard regression model was conducted for univariate and multivariate survival analysis. The student’s *t*-test was performed to compare variables between two groups and multigroup analysis of variance (ANOVA) was utilized for multigroup comparisons. Association between the expression levels of DCUN1D5, YY1, STAT1 and ELF1 was assessed by Spearman rank correlation coefficients. A two-sided *P* value of less than 0.05 was considered statistically significant. All statistical analyses were performed by SPSS 20.0 (IBM) and GraphPad Prism 7.0 (GraphPad). Each experiment was repeated 3 times and presented as the means ± SD (standard deviation).

## Results

### DCUN1D5 is upregulated in TNBC tissue and associated with poor prognosis

**Fig. 1:**
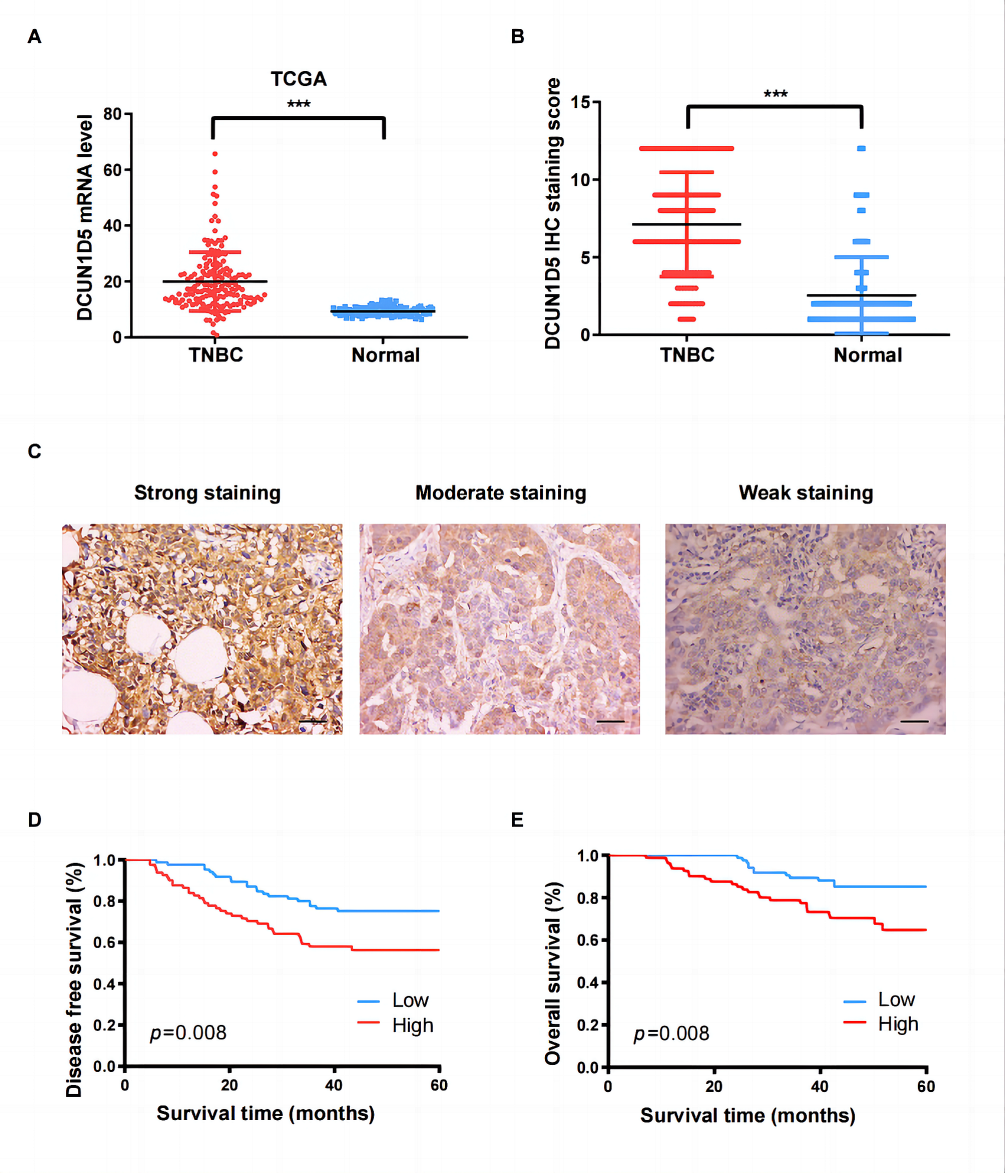
DCUN1D5 is overexpressed in TNBC and significantly associated with poor prognosis. **A** The mRNA expression of DCUN1D5 in TNBC and normal breast tissues was detected by TCGA database. **B** The immunohistochemistry (IHC) scores of DCUN1D5 in tumor and adjacent normal tissues for 166 triple-negative breast cancer patients. **C** Representative IHC images of different DCUN1D5 expression in TNBC tumor tissues (×200). Scale bar: 50 μm. **D, E** Kaplan-Meier analysis for disease-free survival (DFS) and overall survival (OS) with different DCUN1D5 expression in TNBC patients. *** *p* < 0.001

To elucidate the expression pattern of DCUN1D5 in TNBC, we firstly explored DCUN1D5 mRNA level based on TCGA dataset and identified that DCUN1D5 was highly expressed in TNBC tumor tissues than in normal breast tissues (*p* < 0.001, Fig. 1A). Then, IHC staining assay was conducted by 166 pairs of TNBC patient specimens. It was indicted that DCUN1D5 protein level was also significantly elevated in the TNBC tumor tissues than in the corresponding adjacent normal tissues (Fig. 1B, C). Clinicopathological analysis revealed that high DCUN1D5 expression was positively correlated with lymph node metastasis (*p* = 0.014) and lymphovascular invasion (*p* = 0.020) (Table 1). Additionally, patients in high DUN1D5 group had a significantly poorer disease-free survival (DFS) and overall survival (OS) compared to those in low DCUN1D5 group by Kaplan-Meier analysis (Fig. 1D, E). Univariate and multivariate cox analyses demonstrated that DCUN1D5 expression was an independent prognostic factor for both DFS and OS (HR = 1.87, 95%CI = 1.08–3.25, *p* = 0.026 and HR = 2.27, 95%CI = 1.13–4.57, *p* = 0.022, respectively, Table 2). These data suggest that DCUN1D5 has the potential clinical value as a novel diagnostic and prognostic biomarker for patients with triple-negative breast cancer.

**Table 1:** Correlations of clinicopathological features with DCUN1D5 expression for triple-negative breast cancer patients

Variables	All patients		Low DCUN1D5	High DCUN1D5	*p* value^a^
	*n* = 166		*n* = 85	*n* = 81	
	No.	(%)	No.	No.	
Age at diagnosis (years)					0.865
≤50	79	47.6	41	38	
>50	87	52.4	44	43	
Tumor size					0.133
≤2 cm	65	39.2	38	27	
>2 cm	101	60.8	47	54	
Lymph node metastasis					0.014
No	96	57.8	57	39	
Yes	70	42.2	28	42	
Tumor Grade					0.578
I + II	60	36.1	29	31	
III	106	63.9	56	50	
Lymphovascular invasion					0.020
No	103	62.0	60	43	
Yes	63	38.0	25	38	

^a^ The *p* value was calculated among all groups by the Chi-square test

**Table 2:** Univariate and multivariate cox proportional hazard model for disease free survival (DFS) and overall survival (OS) for TNBC patients

Variables	Univariate analysis				Multivariate analysis			
	DFS		OS		DFS		OS	
	HR (95% CI)	*P* ^a^	HR (95% CI)	*P* ^a^	HR (95% CI)	*P* ^a^	HR (95% CI)	*P* ^a^
Age (years)								
≤ 50	Reference		Reference					
> 50	0.96 (0.57–1.61)	0.865	1.28 (0.67–2.45)	0.464				
Tumor size								
≤ 2 cm	Reference		Reference					
> 2 cm	1.46 (0.83–2.56)	0.185	1.43 (0.72–2.84)	0.311				
Lymph nodes metastasis								
No	Reference		Reference		Reference		Reference	
Yes	1.98 (1.16–3.36)	0.026	2.16 (1.12–4.17)	0.022	1.75 (1.02–3.01)	0.042	1.86 (0.95–3.62)	0.069
Grade								
I + II	Reference		Reference					
III	1.38 (0.78–2.44)	0.164	1.61 (0.78–3.33)	0.198				
Lymphovascular invasion								
No	Reference		Reference					
Yes	1.52 (0.90–2.56)	0.121	1.50 (0.78–2.86)	0.223				
DCUN1D5 expression								
Low	Reference		Reference		Reference		Reference	
High	2.09 (1.22–3.59)	0.008	2.55 (1.28–5.08)	0.008	1.87 (1.08–3.25)	0.026	2.27 (1.13–4.57)	0.022

Abbreviation: HR: hazard ratio; CI: confidence interval

^a^ The *P* value was adjusted by the univariate Cox proportional hazard regression model

### DCUN1D5 depletion suppresses proliferation, migration, invasion and induces apoptosis of TNBC cells in vitro

**Fig. 2:**
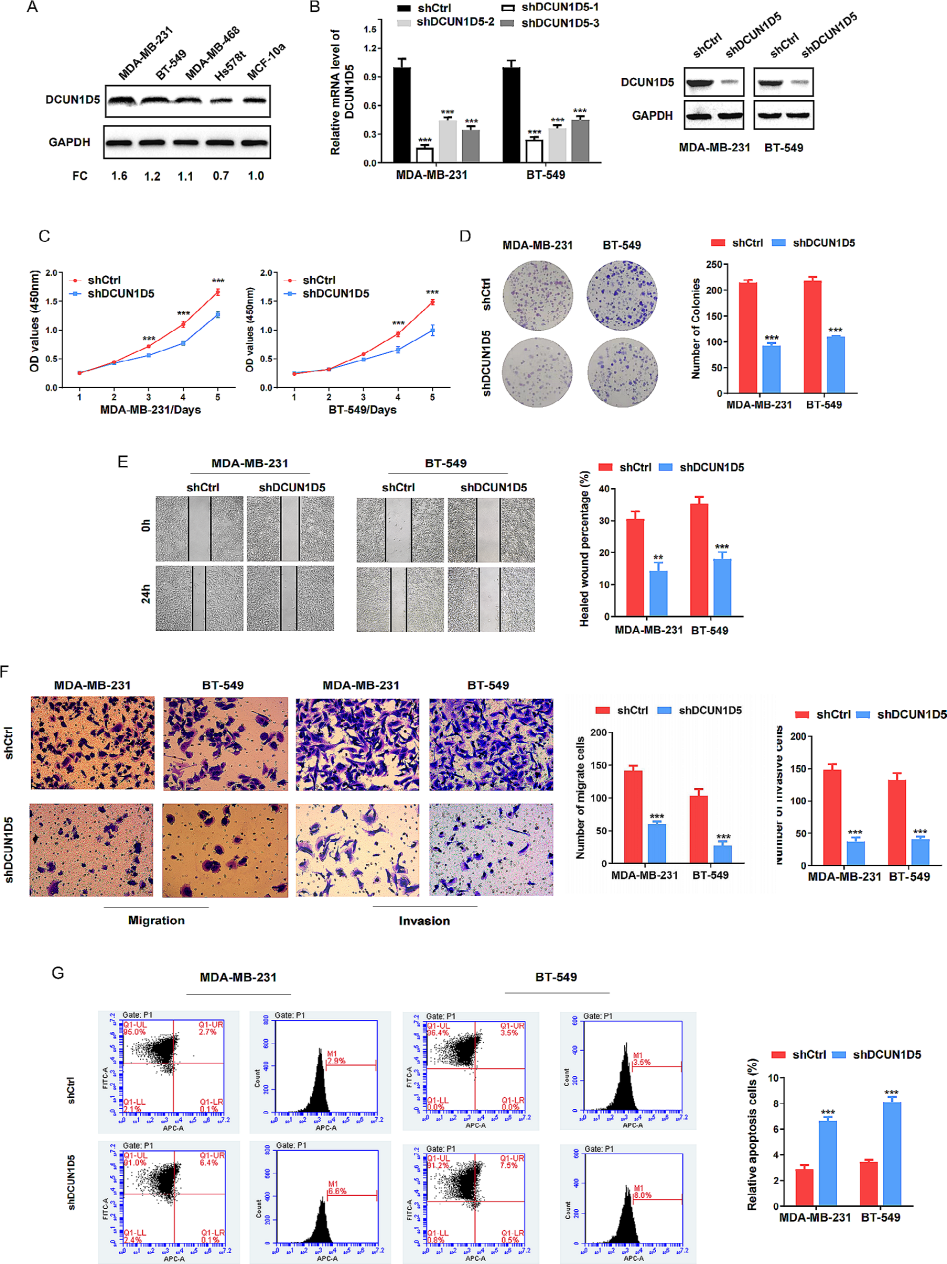
Depletion of DCUN1D5 suppresses cell proliferation, migration, invasion and induces apoptosis of TNBC cells in vitro. **A** The protein expression of DCUN1D5 in TNBC and non-tumorigenic breast epithelial cell line was detected by western blot. **B** The depletion efficiency of shDCUN1D5 was analyzed by qRT-PCR and western blot in MDA-MB-231 and BT-549 cells. **C, D** Cell proliferation and colony formation were assessed by CCK8 and colony formation assay. **E, F** Cell migration and invasion ability was determined by wound healing, transwell migration and invasion assay. **G** Cell apoptosis analysis of MDA-MB-231 and BT-549 cells was evaluated by flow cytometer. FC, fold-change. ** *p* < 0.01, *** *p* < 0.001

To investigate the biological function of DCUN1D5 in vitro, we firstly analyzed the DCUN1D5 expression spectrum in MDA-MB-231, BT-549, MDA-MB-468, Hs578T TNBC cell lines and non-tumorigenic breast epithelial cell line (MCF-10 A). As shown in Fig. 2A, DCUN1D5 was highly expressed in MDA-MB-231 and BT-549 cell lines which were selected for subsequent studies. Both cell lines were infected with DCUN1D5-specific shRNA and the corresponding negative control. The shRNA (shDCUN1D5-1) with higher interference efficiency was confirmed by western blotting (Fig. 2B). A significant decrease in the proliferative and cloning ability was detected of DCUN1D5 knockdown cells by the CCK8 and colony formation assays (Fig. 2C, D). Next, we sought to determine the potential effect of DCUN1D5 in TNBC metastasis. Wound healing, transwell migration and invasion assays demonstrated that knockdown of DCUN1D5 markedly attenuated the migration and invasion ability of both MDA-MB-231 and BT-549 cells (Fig. 2E, F). Furthermore, we also observed dramatically increased apoptotic cells in shDCUN1D5 group compared with that in shCtrl group (Fig. 2G). Collectively, these results provide evidence that DCUN1D5 could suppress proliferation, migration, invasion and induce apoptosis in TNBC cells.

### Overexpression of DCUN1D5 promotes proliferation, migration and invasion of TNBC cells in vitro

**Fig. 3:**
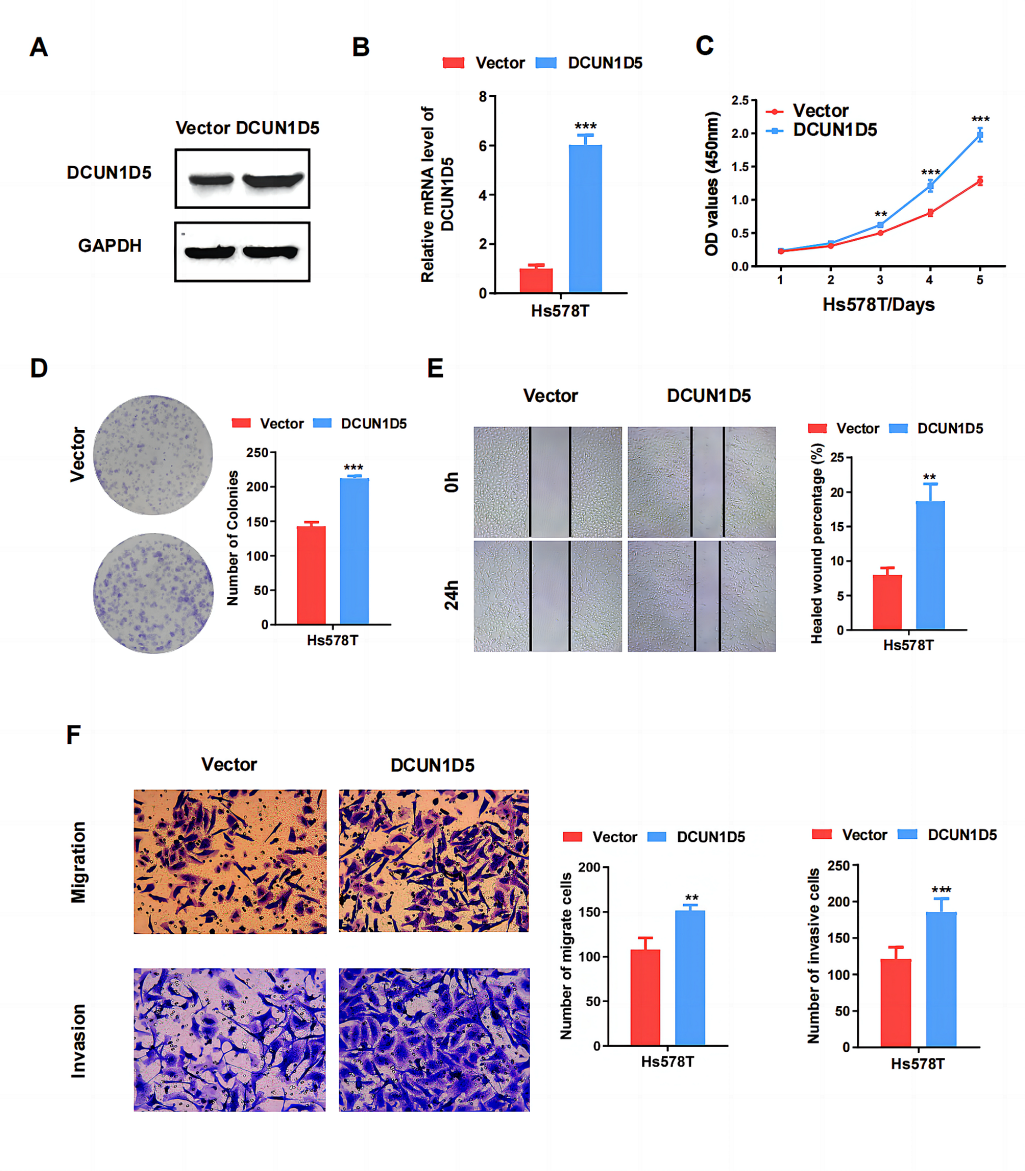
Overexpression of DCUN1D5 promotes cell proliferation, migration and invasion of TNBC cells in vitro. **A, B** The mRNA and protein expression of DCUN1D5 in Hs-578T cells was assessed by qRT-PCR and western blot. **C, D** Cell proliferation and colony formation were analyzed by CCK8 and colony formation assay. **E, F** Cell migration and invasion ability was determined by wound healing, transwell migration and invasion assay

To further address the role of DCUN1D5 in TNBC progression, we generated stable DCUN1D5 overexpressing Hs578T cell lines (Fig. 3A, B). Functionally, DCUN1D5 overexpression significantly promoted the proliferative and cloning potential of HS578T cells (Fig. 3C, D). The migratory and invasive ability was also enhanced in DCUN1D5 overexpressing cells (Fig. 3E, F). These data indicate that overexpression of DCUN1D5 could promote proliferation, migration and invasion of TNBC cells.

### DCUN1D5 promotes TNBC growth and metastasis in vivo

**Fig. 4:**
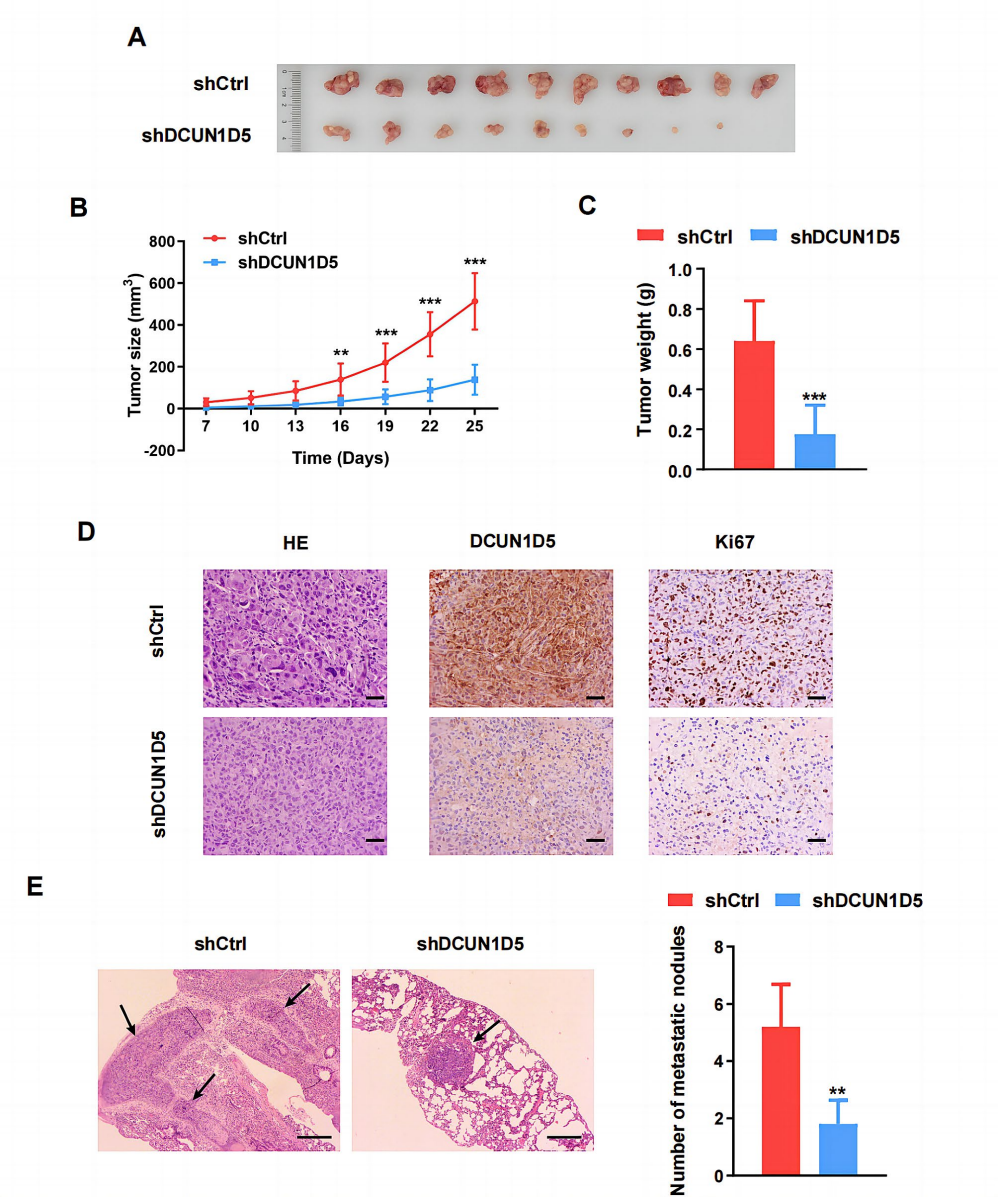
DCUN1D5 promotes TNBC tumor growth and metastasis in vivo. **A** Images of xenograft tumors from groups of BALB/c-nude mice 25 days after the subcutaneous injection of stable DCUN1D5 knockdown MDA-MB-231 cells (shDCUN1D5) or control cells (shCtrl). **B** Tumor volume was calculated according to the formula: (length × width^2^)/2 every 3 days 1 week after injection. **C** Tumor weight in nude mice of shDCUN1D5 and shCtrl group was measured at day 25. **D** Hematoxylin and eosin (HE) staining and immunohistochemistry for DCUN1D5 and Ki67 in xenograft tumors (×200). Scale bar: 50 μm. **E** Representative images of tumor metastasis in lung tissues and the number of metastatic nodules in shDCUN1D5 or shCtrl group. The black arrows indicate pulmonary metastatic nodules (×40). Scale bar: 250 μm. ** *p* < 0.01, *** *p* < 0.001

To investigate the effect of DCUN1D5 on TNBC progression in vivo, MDA-MB-231 cells with stable DCUN1D5 knockdown (shDCUN1D5) and control cells (shCtrl) were injected subcutaneously into the nude mice. As shown in Fig. 4A-C, the tumor volume and weight of xenografts were significantly decreased in the shDCUN1D5 compared with the shCtrl group. Hematoxylin-eosin (HE) and immunohistochemistry staining results also indicated lower percentage of cell mitosis and expression of DCUN1D5 or proliferation marker Ki67 in the shDCUN1D5 group (Fig. 4D). We then examined the effects of DCUN1D5 on TNBC metastasis in vivo. Stable shDCUN1D5 and shCtrl cells were injected into nude mouse via the tail vein. The results revealed that DCUN1D5 knockdown cells formed less metastatic nodules in the lungs compared with the control MDA-MB-231 cells (Fig. 4E). In summary, these findings indicate that DCUN1D5 could promote tumor growth and metastasis of TNBC in vivo.

### DCUN1D5 promotes TNBC progression by activating FN1/PI3K/AKT pathway

**Fig. 5:**
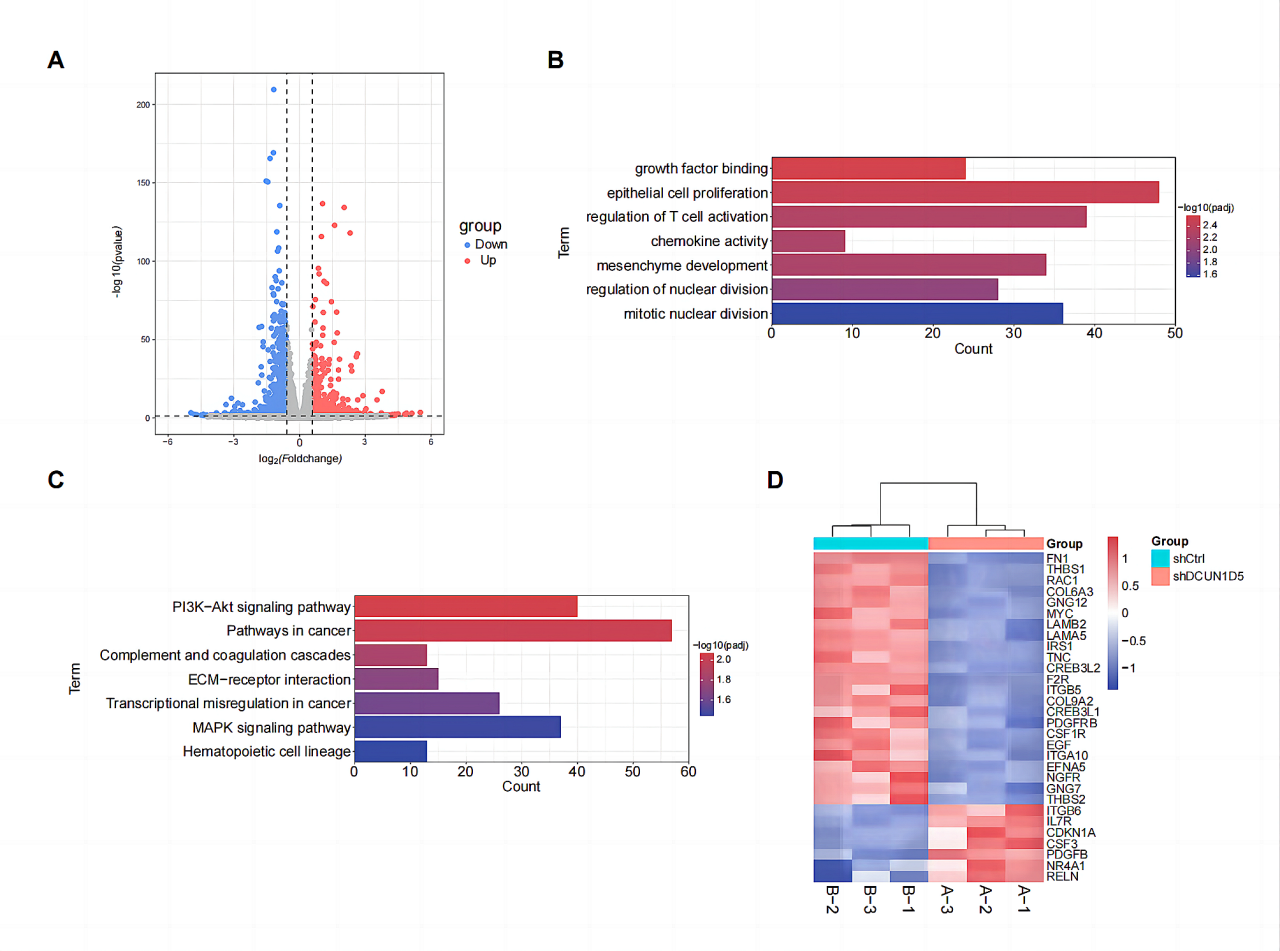
Suppression of DCUN1D5 attenuates PI3K/AKT signaling pathway activity. **A** Volcano plot of differentially expressed genes between DCUN1D5 knockdown (shDCUN1D5) or control cells (shCtrl). **B** The functional category based on gene ontology (GO) term enrichment with different expressed genes between shDCUN1D5 and shCtrl cells. **C** The signaling pathway based on KEGG enrichment analysis. **D** A heatmap of PI3K-AKT signaling pathway-related genes which were differentially expressed between shDCUN1D5 and shCtrl cells

**Fig. 6:**
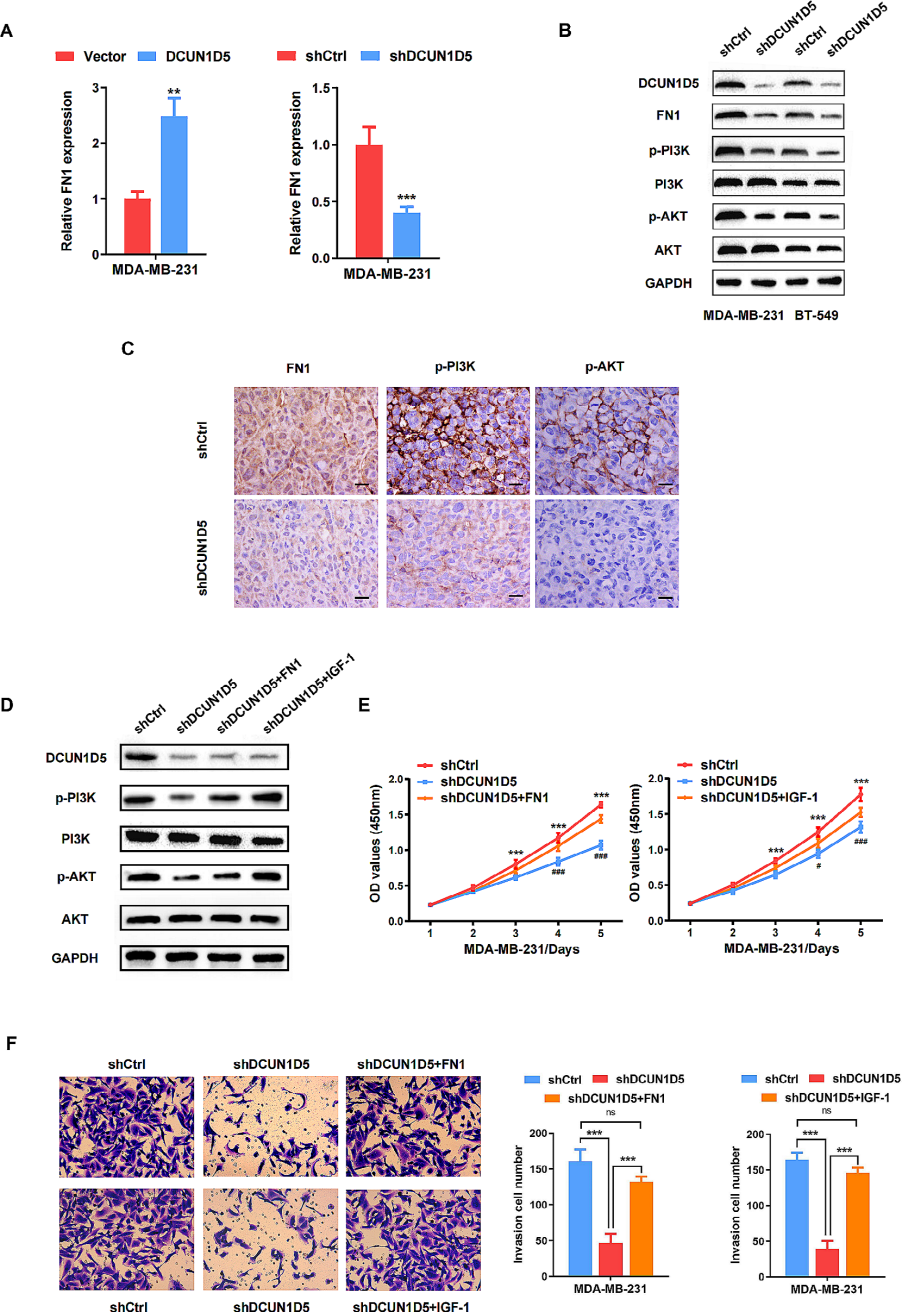
DCUN1D5 promotes TNBC progression by activating FN1/PI3K/AKT pathway. **A** The mRNA expression of FN1 was assessed by qRT-PCR with DCUN1D5 depletion or overexpression. **B** The protein expression of FN1, p-PI3K, PI3K, p-AKT and AKT with DCUN1D5 knockdown was evaluated by western blot. **C** IHC staining of FN1, p-PI3K and p-AKT in xenograft tumors (×400). Scale bar: 25 μm. **D** The protein expression of p-PI3K, PI3K, p-AKT and AKT was assessed by western blot with DCUN1D5 knockdown and FN1 overexpression or IGF-1 treatment. **E, F** Cell proliferation, migration and invasion ability was assessed by CCK-8 and transwell assay with DCUN1D5 knockdown and FN1 overexpression (IGF-1 treatment). ** *p* < 0.01, *** *p* < 0.001, ^#^*p* < 0.05 (shDCUN1D5 vs. shDCUN1D5 + FN1), ^###^*p* < 0.001 (shDCUN1D5 vs. shDCUN1D5 + IGF-1)

Next, RNA-Seq analyses were performed to explore the underlying molecular mechanism by which DCUN1D5 contributes to TNBC progression. A total of 1626 genes, including 630 upregulated genes and 996 downregulated genes (|Fold change| >1.5, *p*adj < 0.05), were differentially expressed in DCUN1D5 knockdown (shDCUN1D5) and control cells (shCtrl) cells (Fig. 5A, Table S3). Functional categories based on gene ontology (GO) confirmed that the terms of growth factor binding, epithelial cell proliferation and regulation of T cell activation were significantly enriched (Fig. 5B). Moreover, KEGG analysis indicated that PI3K/AKT signaling pathway was a dominant component in the enriched pathways (Fig. 5C). The differential gene expression signatures of the PI3K/AKT pathway was shown in Fig. 5D. Then, we used quantitative real-time PCR to select and confirm the most possibly related gene Fibronectin 1 (FN1). It was identified that the expression of FN1 could be significantly regulated by DCUN1D5 in TNBC cells (Fig. 6A). FN1 is one important member of the glycoprotein family and has been proved to regulate PI3K/AKT pathway in numerous tumors [17–20]. Therefore, western blotting was performed to evaluate the protein expression of FN1, p-PI3K, PI3K, p-AKT and AKT with DCUN1D5 knockdown. The results indicated that DCUN1D5 inhibition significantly decreased the expression of FN1, as well as the phosphorylation of PI3K and AKT (Fig. 6B). Moreover, we examined the expression of FN1, p-PI3K and p-AKT in xenograft tumors by IHC staining. Consistent with the western blotting results, the expression of FN1, p-PI3K and p-AKT were significantly downregulated in the xenograft tumors with DCUN1D5 knockdown (Fig. 6C). Meanwhile, rescue experiments were conducted to confirm whether DCUN1D5 exerted its effect in TNBC by FN1/PI3K/AKT activation. An activator of PI3K/AKT signaling (insulin-like growth factors-1, IGF-1, 200ng/ml) was applied in the rescue assay. As shown in Fig. 6D, overexpression of FN1 or IGF-1 treatment restored the expression of phosphorylation PI3K and AKT, and the decreased proliferative and invasive ability induced by DCUN1D5 knockdown could also be rescued (Fig. 6E, F). Taken together, these data demonstrate that DCUN1D5 could promote TNBC progression by activating FN1/PI3K/AKT pathway.

### YY1 activates DCUN1D5 transcription in TNBC cells

**Fig. 7:**
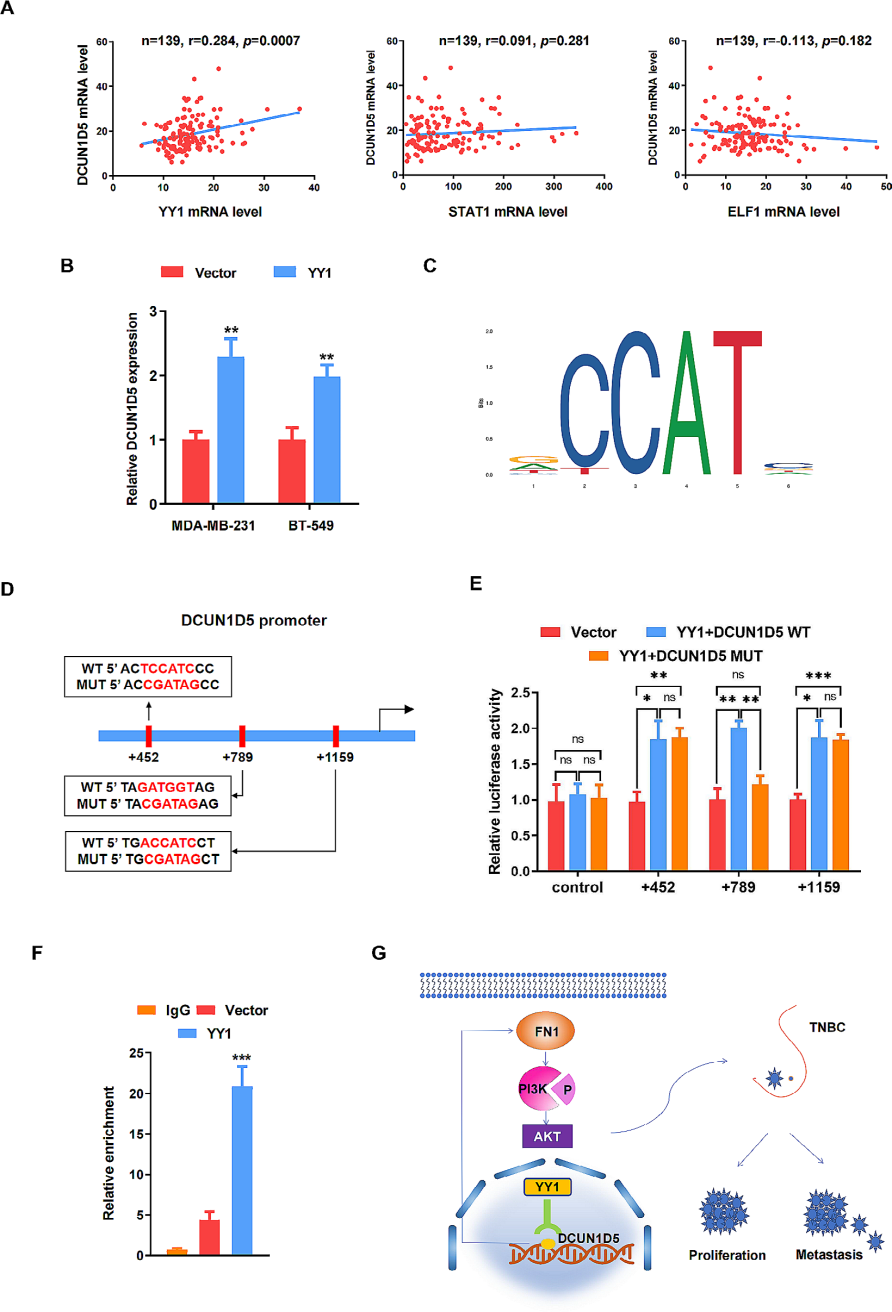
YY1 activates DCUN1D5 transcription in TNBC cells. **A** The correlation between YY1, STAT1, ELF1 and DCUN1D5 mRNA expression was evaluated by Spearman’s correlation analysis based on TCGA database. **B** DCUN1D5 mRNA expression level was identified by qRT-PCR with overexpression of YY1. **C** The binding site of YY1 and DCUN1D5 was predicted by JASPAR database. **D, E** Dual-luciferase reporter assay was conducted by co-transfection of the DCUN1D5 promoter fragment (WT or MUT) and YY1 overexpression construct. **F** ChIP assay was performed to determine YY1 could bind to the DCUN1D5 promoter. **G** A Schematic diagram showing YY1 mediated DCUN1D5 transcriptional activation promotes triple-negative breast cancer progression by targeting FN1/PI3K/AKT pathway. ** *p* < 0.01, *** *p* < 0.001

To further elucidate the molecular mechanism of DCUN1D5 in TNBC progression, we used JASPAR database (http://jaspar.genereg.net/) [21] and PROMO software (http://alggen.lsi.upc.es/cgi-bin/promo_v3/promo/promoinit.cgi?dirDB=TF_8.3) [22, 23] to screen for the potential transcription factor which could regulate DCUN1D5 expression. Three candidates including YY1, STAT1 and ELF1 were selected for subsequent analysis. The expression of these three transcription factors and DCUN1D5 were then evaluated by spearman’s correlation analysis for TNBC samples in TCGA database. Especially, only YY1 showed a positive correlation with DCUN1D5 (*r* = 0.284, *p* < 0.001, Fig. 7A). Given the essential role of YY1 on TNBC progression [24–27], we speculated YY1 as the upstream transcription factor for DCUN1D5. To validate this speculation, we firstly analyzed DCUN1D5 mRNA expression after transfection with YY1 construct. It was found that overexpression of YY1 significantly enhanced the mRNA expression of DCUN1D5 in both MDA-MB-231 and BT-549 cells (Fig. 7B). Then, JASPAR database was utilized to predict the specific YY1 binding sites on DCUN1D5 promoter region (Fig. 7C, Table S4). We mutated the predictive YY1 binding sites (+ 452, + 789, +1159, Fig. 7D) on DCUN1D5 sequence and the luciferase reporter assay indicated that the transcriptional activity of DCUN1D5 was significantly enhanced after transfection with YY1 construct, whereas the increased luciferase activity was reversed by transfection with the mutated DCUN1D5 sequence (+ 789, Fig. 7E). In addition, ChIP assay was also conducted and demonstrated that YY1 could directly bind to the promoter region of DCUN1D5 (Fig. 7F). Overall, these findings confirm that YY1 could transcriptionally enhance DCUN1D5 expression by binding to its promoter region in TNBC cells.

## Discussion

Triple-negative breast cancer is the most aggressive subtype of breast cancer. Despite recent progress in clinical diagnosis and treatment, the prognosis of TNBC still remains poor, especially in patients with recurrence or distant metastasis [2, 3]. Therefore, identification of novel molecular biomarkers and new treatment targets are urgently needed for patients with TNBC. DCUN1D5 is one important member of DCUN1D family which has been demonstrated to promote neddylation as NEDD8 E3 ligase [6]. Recent study has indicated that DCUN1D5 was linked with multiple cancers, including oral and lung cancer [7, 14]. For breast cancer, it is demonstrated that DCUN1D5 was highly expressed in metastatic breast tumors compared with non-metastatic breast tumors. DCUN1D5 expression was also identified to be elevated in high-metastatic TNBC breast cancers compared to low-metastatic luminal tumors in the TCGA-BRCA and CPTAC (Clinical Proteomic Tumor Analysis Consortium) dataset [15]. However, its functional role and biological mechanisms in TNBC has not been reported yet.

In the present study, we explored the role of DCUN1D5 in the development of triple-negative breast cancer and evaluated its prognostic value for patients with TNBC. DCUN1D5 was found to be highly expressed in TNBC tissues with TCGA dataset and surgical specimens by immunohistochemical (IHC) staining method. The upregulated expression of DCUN1D5 was also identified to be correlated with worse disease-free survival and overall survival for patients with TNBC. Moreover, in vitro and in vivo functional studies indicated that knockdown of DCUN1D5 significantly impeded cell growth, migration and invasion abilities for TNBC cells, whereas DCUN1D5 overexpression promoted these biological process. These data validate that DCUN1D5 plays an important role in the progression of TNBC and could serve as a useful biomarker for the prognosis of patients with TNBC.

We then investigated the underlying mechanisms of DCUN1D5 in promoting TNBC progression. By using the RNA-Seq analyses, we revealed that PI3K/AKT signaling pathway was a dominant component in the enriched pathways regulated by DCUN1D5 in triple-negative breast cancer. PI3K/AKT is a key intracellular pathway which participates in various biological processes, including cellular metabolism, growth and metastasis [28]. Numerous studies have showed that the activation of PI3K/AKT pathway is closely correlated with the progression and drug resistance of triple-negative breast cancer [29–32]. Our study reported for the first time that DCUN1D5 could affect the PI3K/AKT signaling pathway in TNBC, which further supplemented the mechanistic investigation of DCUN1D5 in cancer. Moreover, we also identified Fibronectin 1 (FN1) as a key downstream target for DCUN1D5. FN1 is one important member of the glycoprotein family and could act as a positive regulator of the PI3K/AKT pathway [17, 19, 33, 34]. In our study, we demonstrated that DCUN1D5 inhibition significantly decreased the expression of FN1, as well as the phosphorylation of PI3K and AKT in TNBC cells. Rescue assay indicated that overexpression of FN1 or IGF-1 (PI3K/AKT signaling activator) treatment could restore phosphorylation of PI3K and AKT, and the decreased proliferative and invasive ability of MDA-MB-231 cells induced by DCUN1D5 knockdown could also be rescued. Taken together, these results confirm that DCUN1D5 promotes TNBC progression through activation of the FN1/PI3K/AKT pathway.

To identify the upstream transcriptional regulation for DCUN1D5 in triple-negative breast cancer, we used JASPAR database and PROMO software to screen for the potential transcriptional factors. Three candidate transcription factors including YY1, STAT1 and ELF1 were selected for subsequent analysis. The expression of these three transcription factors and DCUN1D5 were then assessed by spearman’s correlation analysis for TNBC samples in TCGA database. It was found that only YY1 showed a positive correlation with DCUN1D5 (*r* = 0.284, *p* < 0.001). Yin Yang 1 (YY1) is a zinc finger DNA-binding protein and a member of the GLI-Krüppel family. Accumulating evidence have suggested that YY1 could participate in numerous biological functions, such as cell proliferation [35–37], apoptosis [38–40], epithelial-mesenchymal transition (EMT) [41, 42] and drug resistance [43–45]. For triple-negative breast cancer, YY1 has also been identified as a critical promoter for tumor proliferation and invasion [25, 26]. In the present study, our data demonstrated the transcriptional activity of DCUN1D5 was significantly enhanced after transfection with YY1 construct in dual-luciferase reporter assay. By utilizing JASPAR database, we also predicted the specific YY1 binding sites on DCUN1D5 promoter region, and the YY1-enhanced promoter activities were abolished by transfection with the mutated DCUN1D5 sequence. In addition, ChIP-qPCR assay indicated that YY1 could directly regulate DCUN1D5 transcription by binding to its promoter. Altogether, these findings confirm the ability of YY1 to bind to the DCUN1D5 promoter and DCUN1D5 could act as a downstream effector for YY1 to promote TNBC progression.

## Conclusions

YY1-enhanced DCUN1D5 expression could promote TNBC progression by FN1/PI3K/AKT pathway and DCUN1D5 might be a potential prognostic biomarker and therapeutic target for TNBC treatment. The findings of this study provide novel insights of DCUN1D5 in the progression of TNBC and suggest a new theoretical basis for the prevention and treatment of patients with triple-negative breast cancer.

### Electronic supplementary material

Below is the link to the electronic supplementary material.

Supplementary Material 1

Supplementary Material 2

Supplementary Material 3

Supplementary Material 4

Supplementary Material 5

## Data Availability

No datasets were generated or analysed during the current study.

## Abbreviations

ChIP: Chromatin immunoprecipitation
CPTAC: Clinical Proteomic Tumor Analysis Consortium
DCUN1D5: Defective in cullin neddylation 1 domain containing 5
DFS: Disease-free survival
EMT: Epithelial-mesenchymal transition
ER: Estrogen receptor
FN1: Fibronectin 1
GO: Gene ontology
HE: Hematoxylin-eosin
HER2: Human epidermal growth factor 2
IHC: Immunohistochemical
OS: Overall survival
PONY: Potentiating neddylation
PR: Progesterone receptor
SCCRO1-5: Squamous cell carcinoma related oncogene 1–5
ShRNA: Short hairpin RNA
TCGA: The Cancer Genome Atlas
TNBC: Triple-negative breast cancer
UBA: Ubiquitin-associated
YY1: Yin Yang 1
