# Supplementary material for: YY1 mediated DCUN1D5 transcriptional activation promotes triple-negative breast cancer progression by targeting FN1/PI3K/AKT pathway
Source: Biol Direct. 2024 Jun 3;19:42. doi: 10.1186/s13062-024-00481-2 (PMC11145835; doi:10.1186/s13062-024-00481-2)
Supplement: Supplementary file 1 — Supplementary Material 1 [file 13062_2024_481_MOESM1_ESM.ppt]

## Slide 1
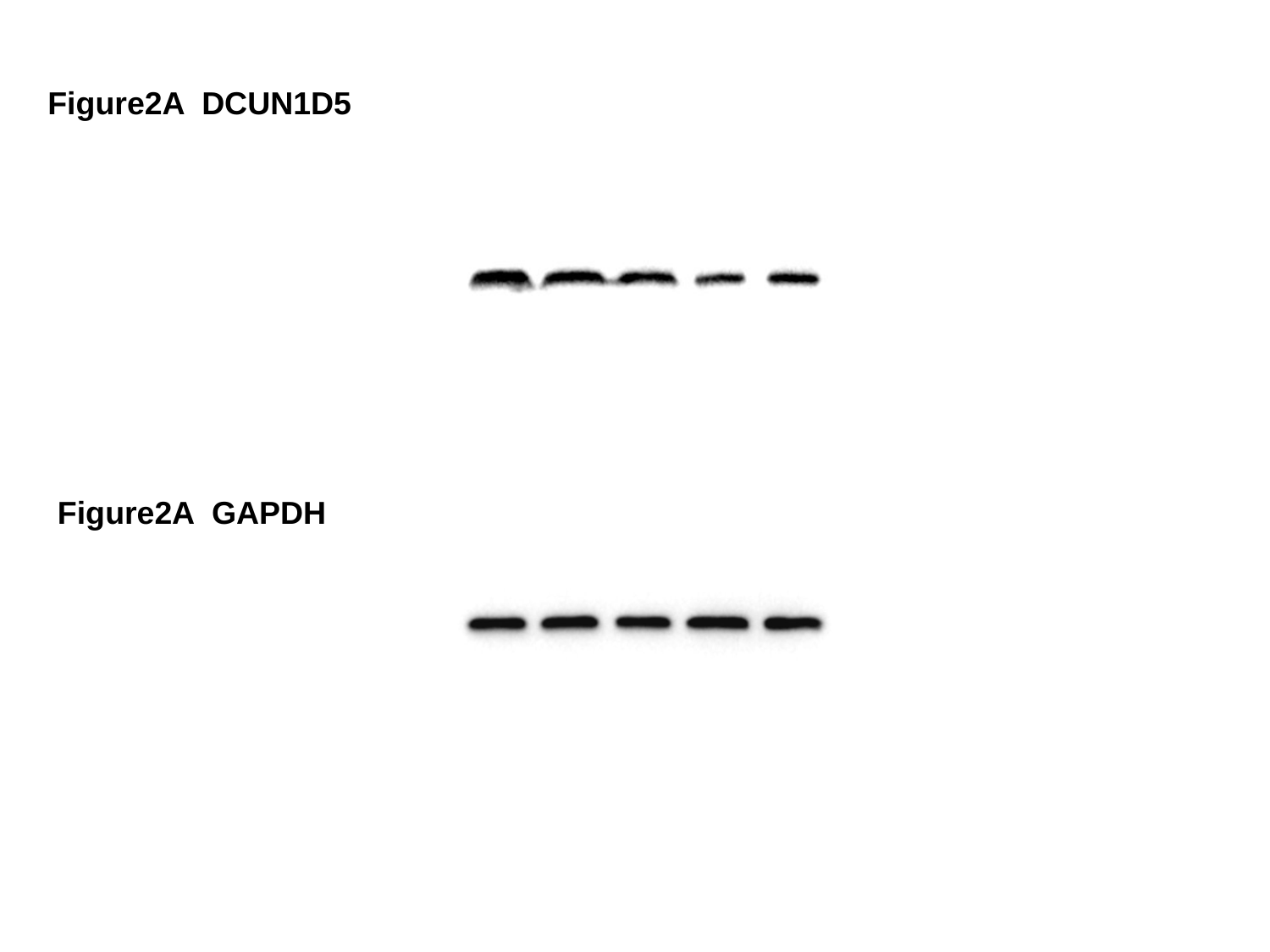

Figure2A DCUN1D5
Figure2A GAPDH

## Slide 2
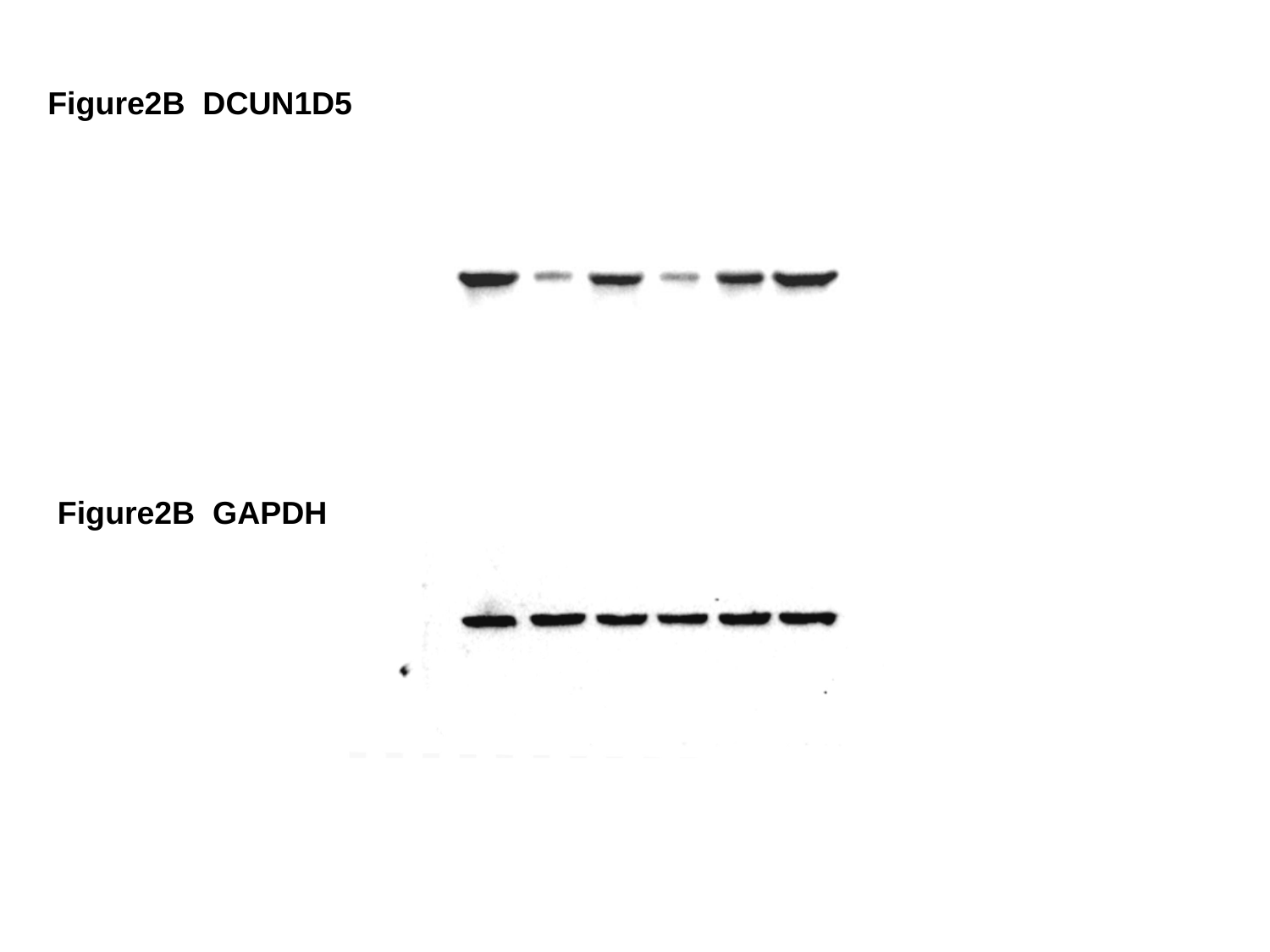

Figure2B DCUN1D5
Figure2B GAPDH

## Slide 3
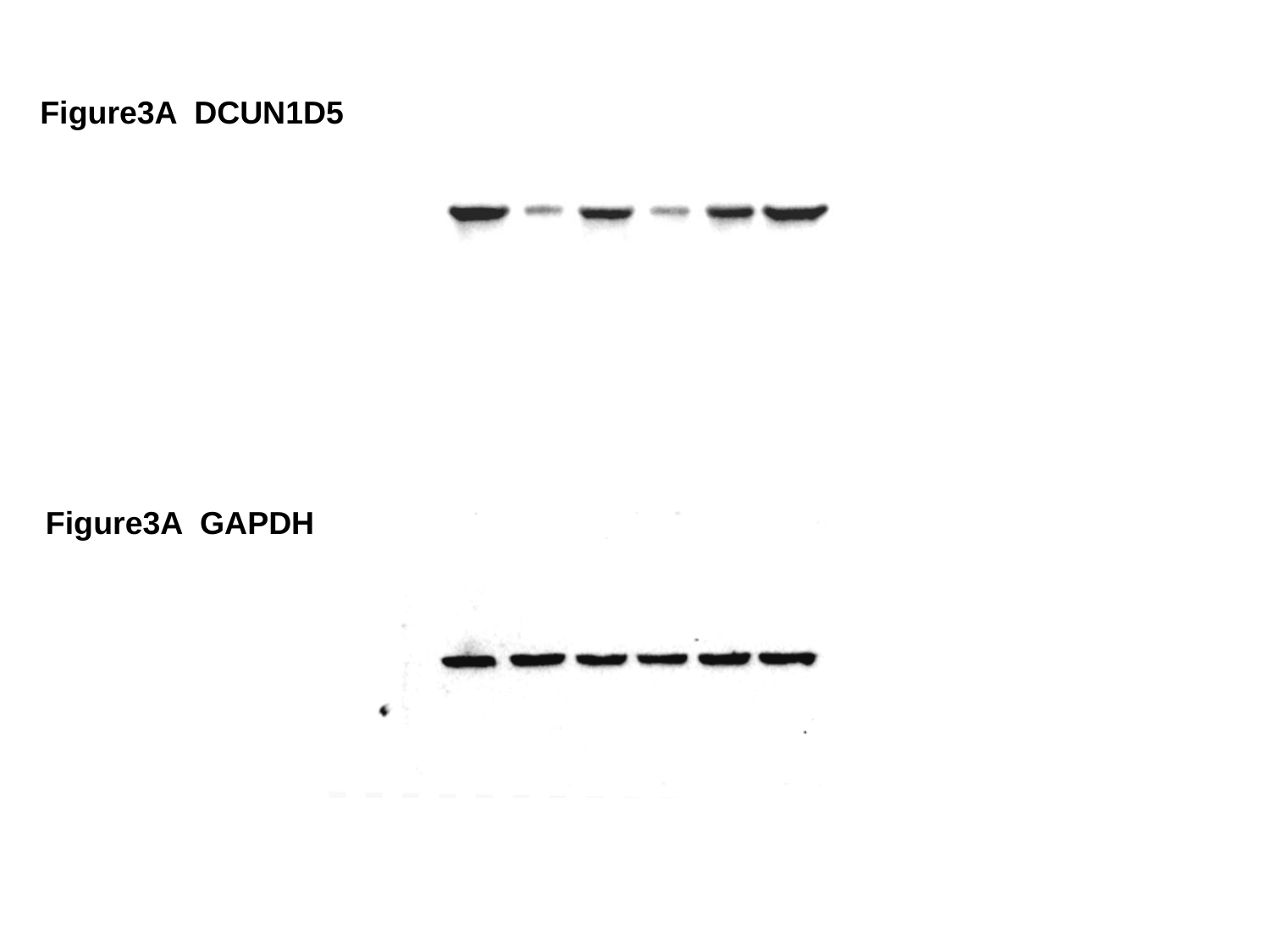

Figure3A DCUN1D5
Figure3A GAPDH

## Slide 4
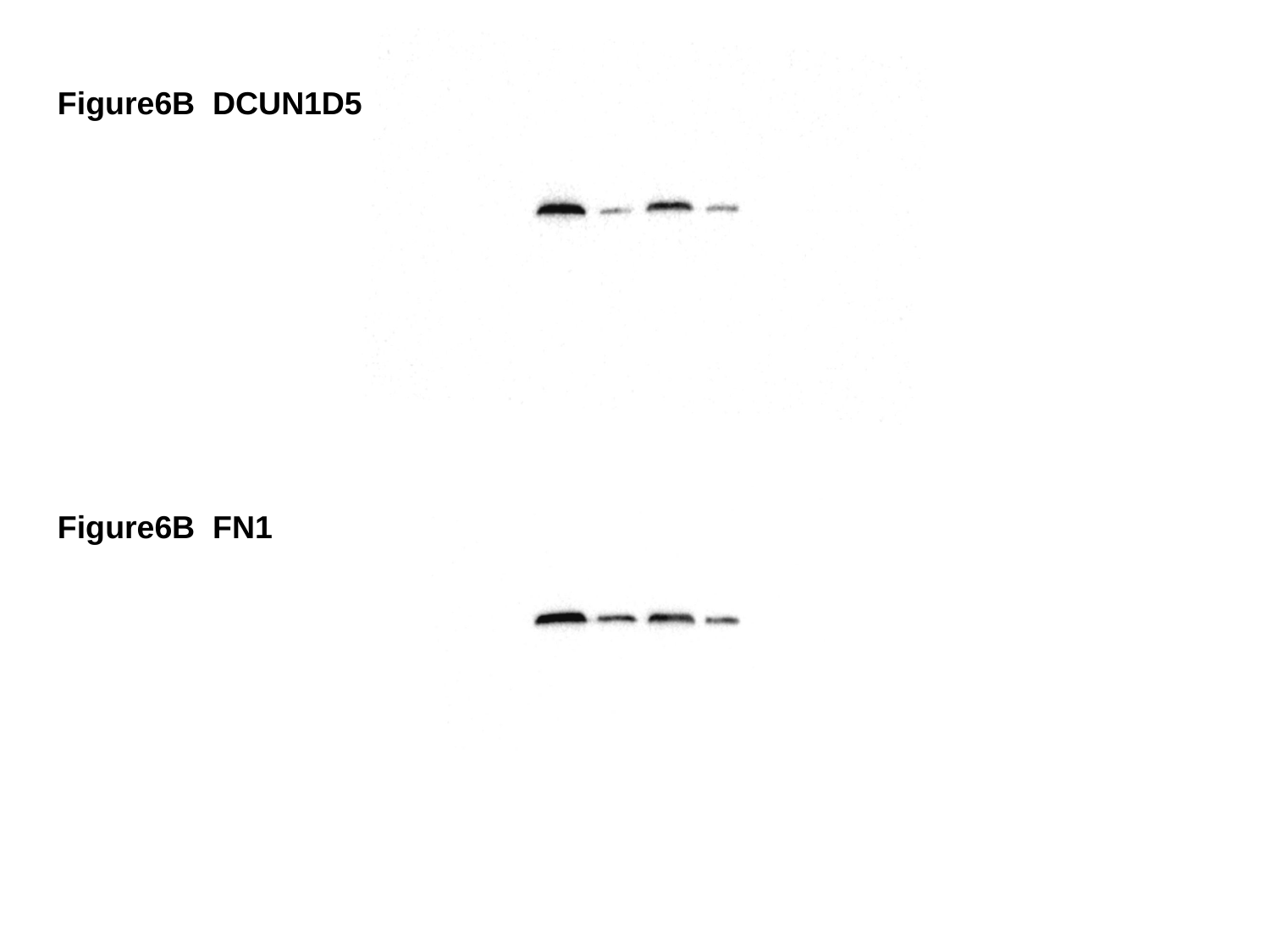

Figure6B DCUN1D5
Figure6B FN1

## Slide 5
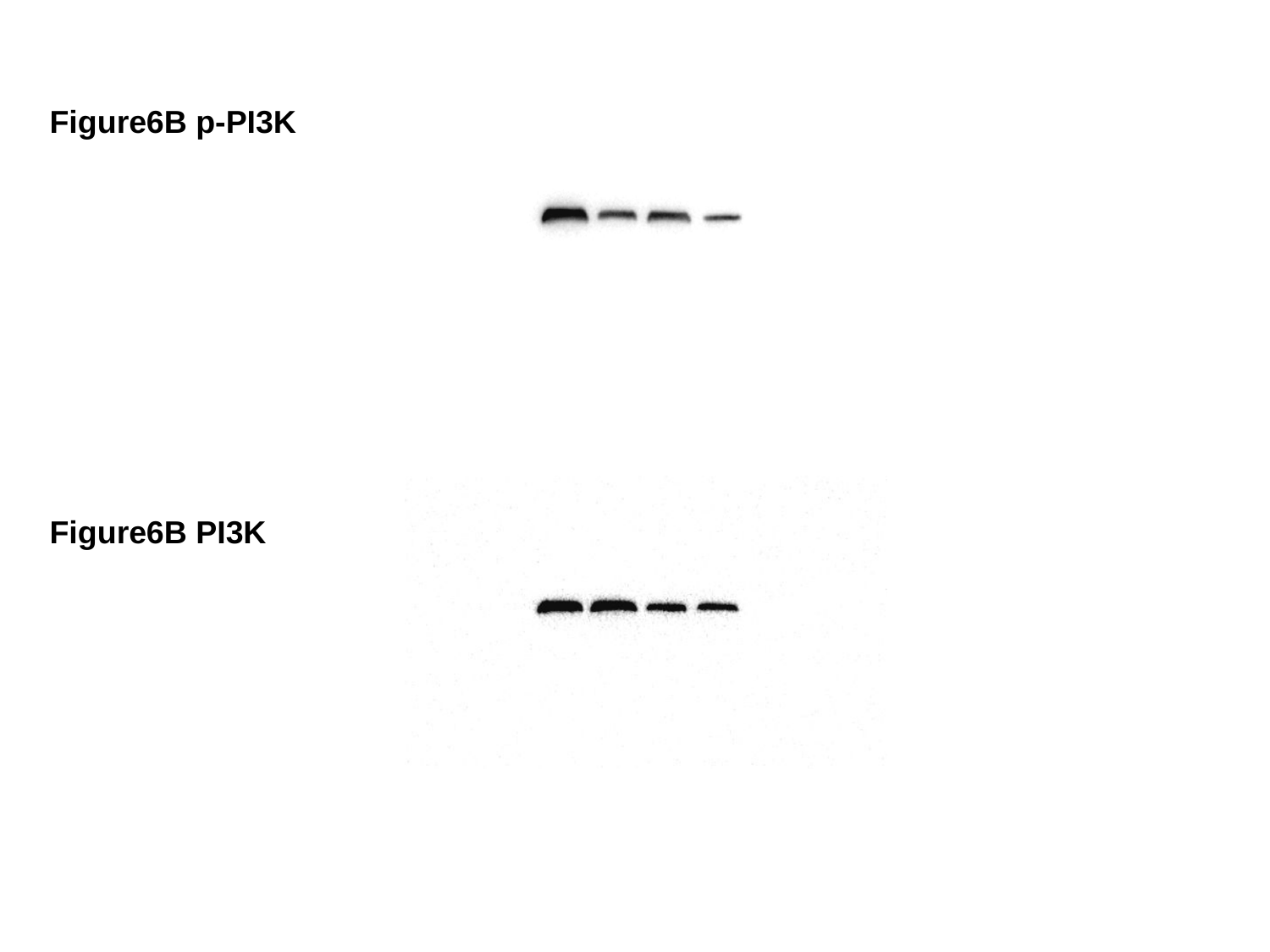

Figure6B p-PI3K
Figure6B PI3K

## Slide 6
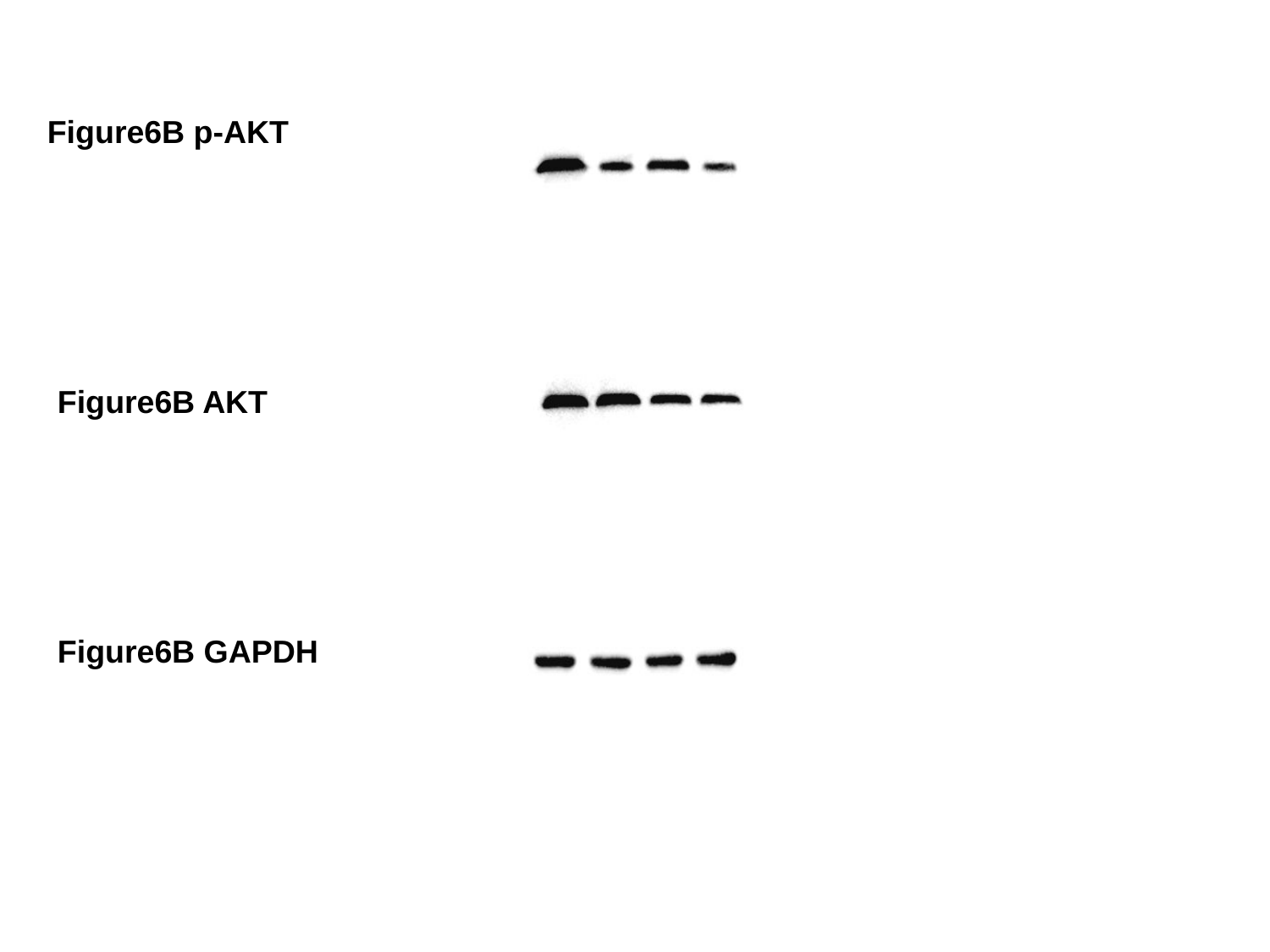

Figure6B p-AKT
Figure6B AKT
Figure6B GAPDH

## Slide 7
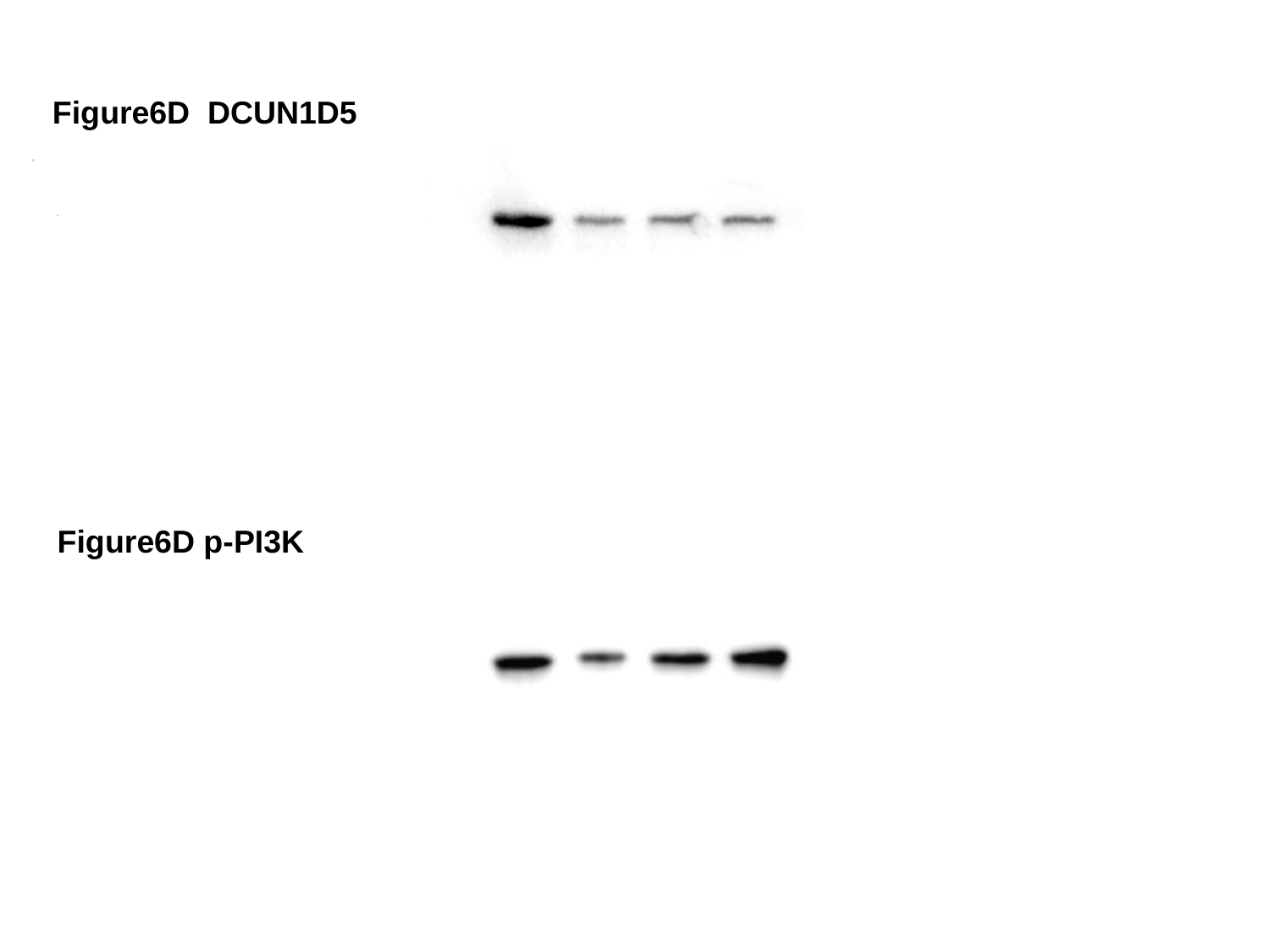

Figure6D DCUN1D5
原始
D
Figure6D p-PI3K

## Slide 8
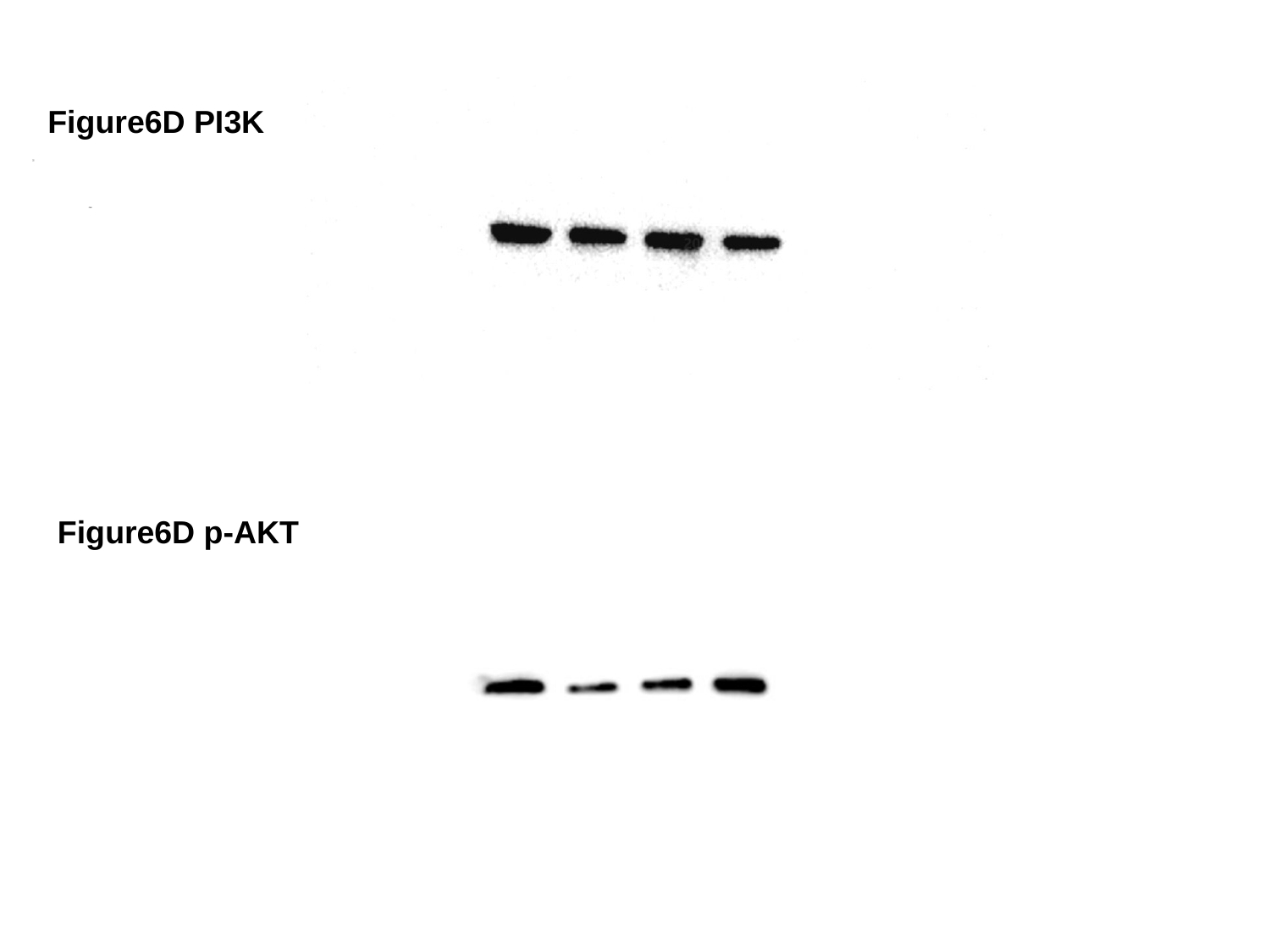

Figure6D PI3K
原始
PI3K
Figure6D p-AKT

## Slide 9
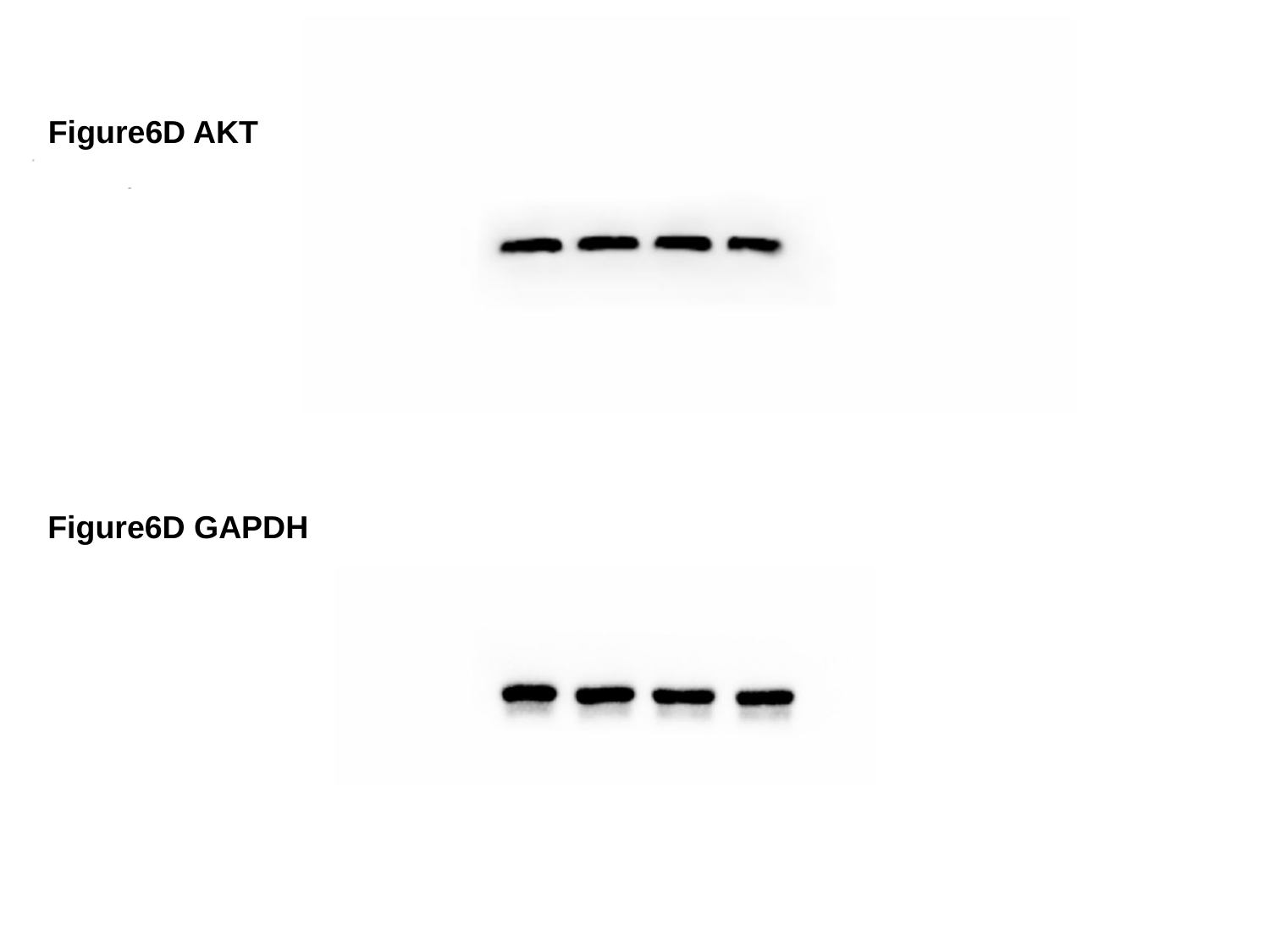

Figure6D AKT
原始
AKT
Figure6D GAPDH
